# Youth-Centered Intervention to Lower the Threshold to Municipal Support and Help Youth Come to Terms with the Dutch Childcare Benefits Scandal: Case Report

**DOI:** 10.2196/94223

**Published:** 2026-09-30

**Authors:** Roel Otto Lutkenhaus, Caspar van Ette

**Affiliations:** 1 Department of Communication Science, Faculty of Behavioural, Management and Social Sciences, University of Twente, Ravelijn (building 10), room 1343, Hallenweg 17, Enschede, Overijssel, 7522 NH, The Netherlands, 31 534895627; 2 New Momentum, Hengelo, Overijssel, The Netherlands; 3 Perron14, Rotterdam, Zuid-Holland, The Netherlands

**Keywords:** formative research, entertainment-education, intervention mapping, theory of change, qualitative research, needs assessment, pilot projects, trust, community participation, feedback

## Abstract

**Background:**

The Dutch childcare benefits scandal (toeslagenschandaal) involved the wrongful labeling of thousands of parents as fraudsters in the childcare allowance system. This led to long-term harm for families and a profound trust crisis in governmental institutions. While the national recovery operation provided financial compensation, municipalities were tasked with offering broader support across life domains to youth who grew up in affected families. Central to this effort was helping them “come to terms” with the scandal by acknowledging, processing, and moving forward from its practical and emotional consequences. Yet, many youths remain hard to reach due to distrust and avoidance of institutional contact.

**Objective:**

This paper describes the development and pretest of a Theory of Change (ToC), grounded in Entertainment-Education (EE) principles, designed to address the municipality of Rotterdam’s challenge of reaching and supporting youth affected by the scandal.

**Methods:**

We used a 2-stage formative approach. First, semistructured interviews with 10 affected youth informed the ToC, segmenting youth by their readiness to come to terms with the benefits scandal, ranging from avoidance to active peer support, and aligning messages to each stage. Second, affected youth and a professional producer-artist cocreated a rap track and music video (“Excuses/Tegenwind” [Apologies/Headwind]) to tell their story and provide inspiration. The video was pretested at a peer-network event, where we evaluated how well it advanced the ToC’s goals through a question-and-answer (Q&A) and moderated group discussion.

**Results:**

The interviews revealed that affected youth experienced the benefits scandal as a disruptive family event, marked by chronic stress, shame, and distrust in governmental institutions. Many described coping through avoidance, ignoring official communication, withdrawing emotionally, and/or relying on self-sufficiency. The ToC prioritized recognition of these experiences, inspiration through peer narratives, and gradual connection to (peer) support. The pretest showed that the cocreated music video aligned with this: youth described the music video as confronting yet validating, noting that it reduced shame and sharing their experiences in their own language. Participants stressed the need for peer-to-peer, low-threshold pathways (eg, WhatsApp [Meta Inc] groups) to sustain engagement.

**Conclusions:**

When governmental institutions face trust issues, formative research is critical to designing effective communication strategies. In this paper, formative research informed a ToC grounded in EE principles, leading to the cocreation of a music video that served as a stepping stone to municipal support by fostering recognition, inspiration, and peer connection. This approach demonstrates how formative research can contribute to interventions that gradually (re)connect with hard-to-reach groups, offering a transferable example for low-trust contexts.

## Introduction

### Background

The Dutch childcare benefits scandal (“toeslagenschandaal”) is a large-scale governance failure in which families, often of non-Dutch descent, were wrongly accused of fraud with state subsidies for childcare and faced aggressive debt collection [1]. In the Netherlands, the benefits system provides state subsidies to help low-income families afford essential expenses, such as childcare, rent, and health insurance. The childcare benefits (“kinderopvangtoeslag”) are part of this system and thereby distinct from general child welfare or universal family allowances; they specifically cofund formal childcare to support parental workforce participation. Families qualify for childcare benefits if their costs exceed specific thresholds while their income remains below set limits. Under this system, families receive monthly advance payments based on estimated income and childcare hours, which are retrospectively settled once actual annual costs and incomes are verified by the tax authorities.

The scandal emerged as authorities deployed automated risk profiling and algorithmic fraud detection that disproportionately targeted low-income families, families with fluctuating childcare hours, and families with non-Dutch names and/or a migration background [2-4]. Targeted families underwent rigid manual checks, where minor administrative errors (eg, a missing signature or invoice) led to full repayment demands, often tens of thousands of euros, rather than minor corrections. Many were also placed on a blacklist, subjecting all future benefit or tax applications to stricter scrutiny.

Thousands of parents, who had relied on these advances to pay for essential services, were forced into severe financial hardship, debt restructuring, and prolonged legal battles to contest decisions [5,6]. The scandal’s impact extended beyond finances: children and youth in affected families experienced disrupted education, developmental setbacks, and long-term psychological harm [7,8].

Following the parliamentary inquiry and subsequent policy decisions, a national recovery operation was introduced that combined financial compensation with broader support. Central to the recovery operation is helping impacted families “come to terms” with the scandal by acknowledging, processing, and moving forward from its practical and emotional consequences. As such, the purpose of the recovery operation is practical and administrative, but also about rebuilding trust in institutions that previously caused harm [9].

The recovery operation also includes a scheme aimed at children and youth from affected families as their developmental and educational trajectories were uniquely derailed by the scandal: the so-called kindregeling (“child arrangement”) [10]. The scheme provides (1) a one-time financial payment between €2000 and €10,000 (US $2295 and US $11,476) by the national government, and (2) access to what is called, “broad support” through municipalities, tailored to the needs of the child or young person [11,12]. For broad support, children and youth can approach their municipality to discuss needs across life domains (eg, finances, housing, family, health, and work), ideally resulting in a plan of action to come to terms with the benefits scandal.

### The Recovery Operation in Rotterdam

With the national government providing financial compensation, and municipalities having to provide “broad support,” municipalities carry an important part of the kindregeling, while facing several challenges in fulfilling this role.

First, municipalities do not always have full visibility on who is eligible for broad support and where these young people are. In the Dutch city of Rotterdam, for example, the municipality lacked precise data on how many families had been impacted by the scandal, let alone clear strategies for reaching them effectively. Second, many young people affected by the benefits scandal have developed distrust in “the system.” Their parents were accused of fraud after applying for support from institutions that were supposed to help them. As a result, some youth avoid governmental support systems and official communication altogether. Third, affected families often feel ashamed and forbid their children to talk about what happened to them. Fourth, even when there is willingness to seek support, access can be unclear or feel unsafe. Support is often organized according to an institutional logic, leading young people between desks and departments and confronting them with waiting times. Meanwhile, youth are dealing with urgent, everyday problems and often prefer to discuss these with a single trusted contact person. Many fear being judged, registered, blamed, or pulled into a bureaucratic process with uncertain benefit.

It is for these reasons that the municipality of Rotterdam, where a proportionally higher figure of families and youth have been impacted by the scandal [13], commissioned a project in 2023 exploring whether a narrative media intervention could help youth “come to terms” with the scandal by reaching youth and lowering the threshold to engage with available municipal support. This case report describes the formative work that followed, leading to a cocreated music video that tells the story of the benefits scandal from a youth’s perspective, and an intervention strategy that leverages the music video to link youth to peer-support. The purpose of this report is to document an innovation-in-context: what we did, how we did it, and what seemed feasible, risky, or necessary for scaling.

## Methods

### Study Design

In January 2023, the Municipality of Rotterdam approached New Momentum [14], a research and consultancy agency specialized in social media and social change to which the first author (RL) is affiliated, and Perron14 [15], an agency focused on government communication in Rotterdam to which the second author (CvE) is affiliated, for guidance on strategies to reach affected youth and link them to municipal support. The commissioning context was municipal recovery work related to the childcare benefits scandal and broader support for affected youth (ie, the kindregeling).

From the start, and as per the preference of the municipality, we proposed to cocreate narrative media with youth, embedding it in pathways that build up to peer contact and ultimately lead to municipal support. We rooted this idea in the Entertainment-Education (EE) strategy, using it as our main framework. EE seeks to use storytelling in popular media formats to influence knowledge, attitudes, and behavior, and to stimulate informal conversations that disseminate information and ideas [16-18]. Thus, EE does not only aim at persuading individuals, but also at triggering conversation, often peer-to-peer, lifting the taboo off topics and shaping social norms. Furthermore, recent advantages in EE focus on leveraging social influence in online social networks to communicate with audiences that have moved beyond mass media, for example, by collaborating with creators influential among target audiences [19,20].

We recognized that our case represents a challenge in a complex system that involves multiple stakeholders and multifaceted issues. To address this complexity, we split our work in 2 formative phases - one to better understand the issue and propose a solution, and one to pretest the proposed solution - that we aligned with the Intervention Mapping (IM) approach [21,22]. This section describes the activities during these phases, how they align with the IM framework (Figure 1).

**Figure 1.**
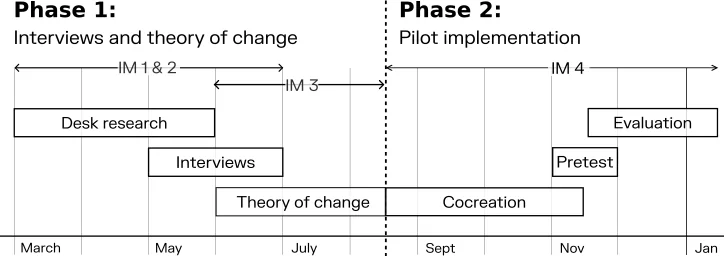
Flowchart showing the activities during the 2 formative phases, and their alignment with the intervention mapping framework.

### Phase 1: Interviews and Theory of Change

The first formative phase, spanning from March to mid-August 2023, contributed to our understanding of the issue (IM step 1: logic model of the problem) by gathering insights through desk-based research and informal stakeholder conversations, and 10 in-depth interviews with affected youth.

### Desk Research

We started the project by conducting desk-based research on the benefits scandal, focusing on the size and scope of the issue in the Rotterdam region. We had informal conversations with municipal officials and impacted families and youths on how young people were impacted, and on how the recovery operation had been organized so far. These activities contributed to a better understanding of the problem, as well as the potential support for different kinds of solutions among the affected youth as well as the municipality.

### Interviews

In May and June 2023, we conducted 10 semistructured interviews with youth affected by the childcare benefits scandal. The interview format was structured around 4 sensitizing concepts that guided the conversations within a constant comparative, inductive approach to analysis [23]. The 4 concepts addressed (1) identity and social environment, (2) the benefits scandal and its consequences, (3) coping and perceived solutions, and (4) future orientation (Table 1). This sequencing was intentional because it allowed participants to define what matters to them in their own terms before discussing sensitive experiences. The interviews were conducted by the first author (RL) and a young journalist (JD), whose credibility and popularity among the target audience helped establish trust during the conversation. The interviews lasted approximately 1 hour and were audio-recorded with consent.

**Table 1.** Overview of the sensitizing concepts that were used during the interviews, including example questions.

Sensitizing concept	Example questions
Identity and social environment	Who are you? Where do you live? What do you enjoy and what not? Who are your parents, siblings, and friends? What did you want to become when you were younger?
The benefits scandal and its consequences	When were you first confronted with the benefits scandal and what did that look like? What were the consequences? How did it influence your self-image and your view of society?
Coping and perceived solutions	What has society done to help you? What do you expect from them? What have you done yourself to deal with it? What would you advise someone in your situation?
The future	Where do you see yourself in the future?

### Data Analysis

The interviews were transcribed and analyzed using the constant comparative method [24]. Themes were developed iteratively within and across interviews, with attention to both recurring patterns and meaningful differences.

To remain close to participant language and to identify important associations, semantic network mapping was used as a supportive technique. To create the semantic network, the interview transcripts were loaded into RStudio (Posit PBC) [25]. We lemmatized the texts using the *Frogr* package [26], which calls the Dutch Frog lemmatizer [27]. Using the New Momentum Analysis Toolkit for R (The New Momentum Foundation) [28], Dutch and English stopwords were removed, and word frequencies were inspected. Based on these frequencies, a semantic network was created around the top 5% most frequent words, linking them to words within the top 15% most frequent words, provided that they cooccurred in at least 25% of the sentences in which the words appeared. In this way, we laid bare semantic patterns across the data set.

### Theory of Change

Based on the insights of the desk research and interviews, we developed a Theory of Change (ToC), which is a theory about how particular activities, resources, and interventions can spark change [29,30]. The ToC described how change was expected to occur (IM step 2: program outcomes and objectives - logic model of change) [31], and, using the Diffusion of Innovations theory [32] as a design lens, segmented audiences and messages based on readiness to come to terms with the benefits scandal. The ToC made assumptions clear and testable by outlining steps and processes through which the intervention was thought to lead to the desired outcomes while identifying design requirements for success [29,33]. Then, we identified behavior change methods that influence the determinants and translated these to practical applications that fit the intervention context (IM step 3: program design) [31]. By mid-August 2023, we presented our findings, the ToC, and a plan for the second formative phase, which comprised the production of a pilot and a pretest to assess whether the assumptions underlying the ToC held up.

### Phase 2: Pilot Implementation

#### Cocreation

The second formative phase took place from mid-August to December 2023 and involved the cocreation of a music track and video titled *“*Excuses/Tegenwind” [Apologies/Headwind] about the benefits scandal from affected youth’s perspective (Multimedia Appendix 1) [34]. Cocreation occurred in the studios of Quardin [35], a youth organization for talent development and was led by Quardin’s producer-artist Vlins, who translated youth stories into a music track and video and played a visible role during the pretest.

Two affected young people collaborated with producer-artists Vlins to translate experiences into lyrics and narrative structure, supported by a professional production team for filming and editing. The process started with an intake conversation to bring youth in and set boundaries around what felt safe to share. This first step was deliberately about relationship building: explaining the process, listening to what participants wanted the track to say about both the past and the future, and agreeing on what would remain private. This was followed by a writing session in which experiences were translated into language that felt natural to the youth, and workable as lyrics. From there, the track was finalized and, with 2 more young people joining, the video clip was filmed, with edits in multiple formats for use on social media.

#### Pretest

The creation of the track and music video was followed by a pretest among 13 affected youth to evaluate how well the video advanced the goals of the ToC and to test the assumptions underlying the ToC, aligning with IM step 4: program production and pretest [31]. The pretest took place during the so-called Flow Your Mind event on November 17, 2023. Flow Your Mind (Het Ondersteuningsteam) [36] is a peer-support network set up by the municipality where affected youth can meet peers, access workshops, and connect to municipal support. The network had been set up well before the start of this project, but the municipality struggled to reach affected youth. The event included a premiere of the music video, followed by a question and answer (Q&A) session with the producer-artist and the 2 youth who had collaborated on the production, and a group discussion with 13 participants moderated by the producer-artist. The purpose was to capture immediate reactions and to assess perceived recognition, emotional safety, usefulness for reaching peers who are not engaged, and preferences for dissemination and follow-up. The Q&A session and group discussion were video recorded. The audio was transcribed and analyzed with the constant comparative method [24], using the stages of the ToC (ie, to what extent does the video contribute to the goals of each stage) as an analytical lens. Quotations were selected to illustrate dominant themes.

#### Evaluation

In December 2023, the pilot findings were presented to the municipality, together with a proposal for a pilot to scale up further. We proposed a small-scale pilot using the music video to reach and engage affected youth through a series of hybrid (online-offline) meetups across the city.

### Ethical Considerations

Under Dutch law, formal research ethics committee review is governed by the Medical Research Involving Human Subjects Act (Wet medisch-wetenschappelijk onderzoek met mensen [WMO]) [37]. This study falls outside the scope of the WMO as it comprises nonmedical, qualitative social research that did not subject participants to physical procedures or impose behavioral interventions infringing on their integrity. Furthermore, this study was commissioned by the Municipality of Rotterdam and conducted by independent commercial agencies (New Momentum and Perron14), that are not legally required to maintain internal ethics review boards for non-WMO research. Therefore, no ethics committee review was sought. The first author’s (RL) affiliation with the University of Twente started after the completion of the project; and, therefore, the university’s institutional ethics policy did not apply to the execution of this study.

Instead, ethical oversight was ensured through strict adherence to GDPR (General Data Protection Regulation) and Dutch data protection guidelines [38], with oversight from the Municipality of Rotterdam’s legal team. A shared responsibility agreement between New Momentum and the Municipality of Rotterdam formalized data stewardship roles (New Momentum and Municipality of Rotterdam; document, May 3, 2023), while New Momentum’s privacy policy and information security framework (aligned with ISO 27001 and NEN 7510) governed technical safeguards (eg, encryption) and organizational measures (eg, training). An organizational Data Privacy Impact Assessment (DPIA), completed in May 2023, audited compliance and risk mitigation, including pseudonymization, restricted access to essential personnel, storage on encrypted servers in the European Union with 2-factor authentication and retention limits for raw files (Legal Specialist Privacy, Law & technology & New Momentum; document, May 1, 2023).

Interview participants were recruited via a WhatsApp (Meta Inc) invitation signed by a peer from the Flow Your Mind network to ensure trust and clarity. The message explicitly stated the purpose of informing municipal support strategies, while emphasizing anonymity, voluntary participation, and the right to withdraw at any stage. At the start of the interviews, participants received verbal confirmation of these principles, including their right to pause or terminate the session and request data deletion after the interview. All participants were 18 years or older and provided explicit consent. Given the potential for distress, interviews were conducted with a stop-and-check approach: when sensitive experiences arose, the interview was paused and participants were explicitly asked whether they felt comfortable continuing and sharing the information. As compensation, participants received a €50 (US $57) cinema voucher.

The 2 youths who cocreated the rap track and the 2 youths who starred in the music video were informed in writing and verbally that the pilot had dual purposes: a peer-focused pretest of the track and video, and potential broader dissemination as part of the intervention. All participants were aged 18 years or older, and no demographic details are reported to protect anonymity. All participants provided explicit consent for their participation, with the right to withdraw or request deletion.

All 13 participants at the Flow Your Mind event were aged 18 years or older and informed via email and verbally about the event’s purpose to evaluate the video’s resonance. They were informed that the event would be video recorded for the Flow Your Mind website [36] and social media page, and that the audio would be used for research purposes. They were assured that videos or stills would not be shared without their permission, and that quotes in the report would be anonymized with no individual attribution. All 13 participants provided explicit consent on entering the event. New Momentum’s DPIA also covered event data, ensuring compliance with GDPR.

## Results

### Phase 1: Interviews and ToC

#### Interviews

##### My Family Against the World

Across interviews, the childcare benefits scandal was described as a prolonged family period marked by stress and instability. The semantic network (Figure 2) aligns with this interpretation. One prominent cluster focuses on family (ie, “moeder” [mother], “vader” [father], “kind” [child], “ouder” [parent], “broer” [brother], “zus” [sister]), supporting the idea that youth primarily narrated the scandal as something they went through as a family, with the “boze buitenwereld” (“boos” [angry] and “buitenwereld” [world out there]) as an external threat. In the interviews, participants linked the scandal to concrete memories: relocation, unopened letters, parental exhaustion, and tension at home.

**Figure 2.**
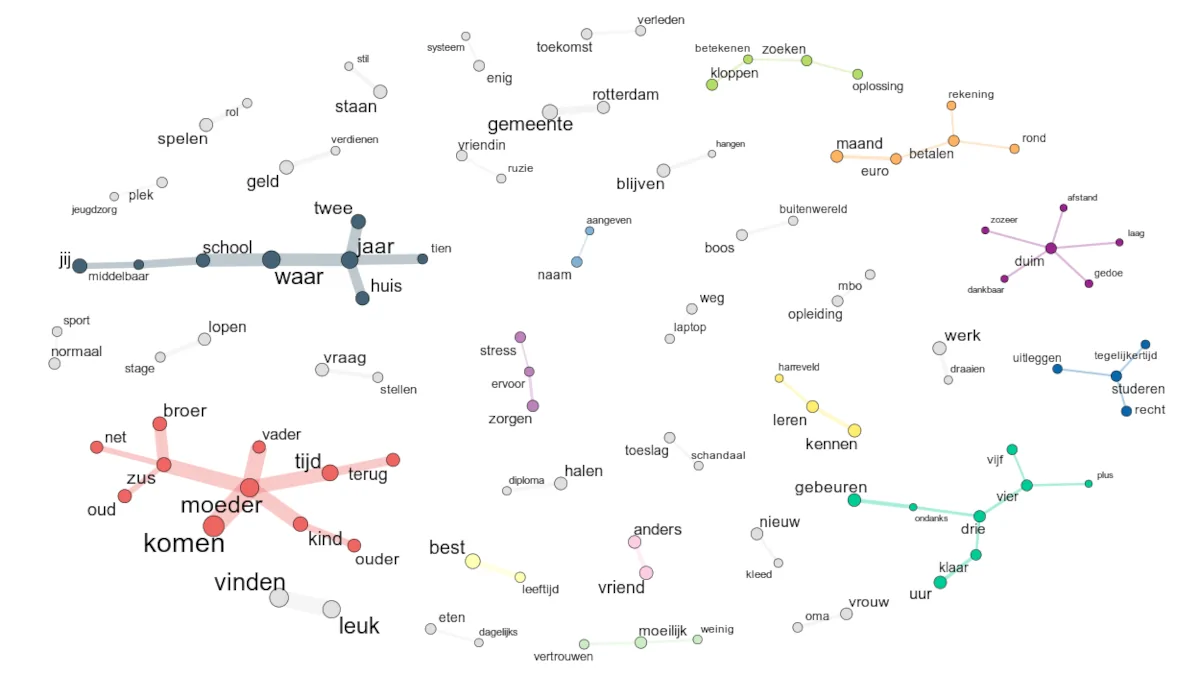
A semantic network showing the most frequently used words during the interviews and their associations. Node and word size reflects frequency, while links represent co-occurrence in sentences.

For example, 1 participant reflected on missing out on basic things during adolescence:

*I have even worn my dad’s shoes at some point… Because I needed new shoes, but we simply could not afford that.*

[P3]

The semantic network reflects narratives of material deprivation with cooccurrence between words such as “eten” (food) and “dagelijks” (daily), kleed (lemmatized version of “kleding,” meaning “clothing”) and “nieuw” (new), and “euro,” “betalen” [to pay], and “rekening” [bill]. It often pushed parents and their children to make ends meet (“geld” [money] and “verdienen” [to make], “rond” [from “rondkomen,” meaning “to make ends meet”], and “maand” [month]).

It also suggests that financial hardship is associated with stress in families (“stress,” “ervoor” [for], and “zorgen” [“to cause,” “to care” and “worries”]), causing tensions (“ruzie” [fight] and “vriendin” [(girl)friend]), and sometimes leading to interference of child protection (“plek” [place] and “jeugdzorg” [child protection]).

Routine acts such as opening the mailbox with the chance of having to see received official correspondence became anxiety triggers. One participant described how relief came only years later:

*I am finally able to open the mailbox without worrying.*

[P2]

In several interviews, youth described how their education was disrupted, how early adulthood became centered on survival, and how a sense of being “behind” persisted even after the financial problems were resolved. This is also reflected in the semantic network by the connection between the words “staan” [standing] and “stil” [still].

##### Why Youth Stay Out of Sight

Youth were aware that something was wrong at home, yet often felt too ashamed to share this with others. Several participants described how others suggested that the government would not impose severe measures without reason, implying that families must have done something wrong. As 1 participant recalled, when trying to explain what had happened:

*Some people said, like… no: that is impossible, man. The government will not screw you over like that.*

[P3]

Such reactions subtly shifted suspicion onto the family. Over time, repeated disbelief led some youth to internalize this guilt. Shame therefore did not only stem from financial problems, but also from the internalized guilt.

For some, this suspicion connected to a broader sense of discrimination. Participant 10 explicitly framed the scandal as “a heavy form of discrimination” and “racism,” and described how exhausting it can be when others still refuse to see it that way:

*When I tell my story, I am often confronted with white people who point out that this is not racism. […] Why do they need to point that out? Why can’t they just listen to me?*.

[P10]

This quote is illustrative of several participants stressing the importance of being able to speak about the benefits scandal “in their own words,” in a form that feels natural and non-institutional.

Many affected youth were deliberately shielded from what was exactly happening. For example, participant 2 described how their mother protected the family from the “boze buitenwereld” (evil world out there; which also is reflected in the semantic network [“boos” and “buitenwereld”]). This delayed understanding of where financial troubles and family tensions came from. Participant 10 only later realized the backdrop of what was experienced as a child:

*Now that I know what was the cause of all of this exactly…I mostly feel happy that this is all behind me.*

[P10]

At the time, participant 10 described, everything “just happened to you,” and the participant did not realize that it was large-scale institutional failure that was behind this.

When talking about how the participants coped with the benefits scandal, the buildup of debts, and subsequent restructuring processes, avoidance emerged as a recurring strategy. At a practical level, this included avoiding correspondence with institutions:

*Then I’d receive letters, but I just pushed it away. I wouldn’t look at them.*

[P10]

This was not simply procrastination, but reflects an association between official communication and escalation. For some, this almost became automatic.

This avoidance often took the shape of what appeared to be a fight-or-flight response: youth described escaping into hobbies (eg, music, manga, and going out), or acting out at school or spending time with “the wrong crowd,” as a way to not think about home. Some participants also described emotional withdrawal or substance use. Participant 3 reflected on a period in which

*I just blocked everything to not turn crazy.*

[P3]

Another participant spoke openly about substance use as a way of managing distress, while others described depressive episodes and worse during peak stress.

In all of these accounts, avoidance was about survival. Blocking emotions, withdrawing, or using substances were short-term regulation strategies. For some participants, avoidance strategies evolved into disengagement with the government altogether. Distrust was rarely expressed as anger, but mostly manifested as lowered expectations:

*I have no expectations. I rely on my own strength.*

[P5]

Having no expectations seemed to be a learned stance among multiple participants, as repeated disappointment led to avoidance of governmental services and self-reliance, with multiple participants stating that they do not apply for benefits again, even though they are aware that they are entitled to them. The semantic network also reflects that affected youth find it difficult to trust again (“vertrouwen” [trust] and “moeilijk” [hard]). This does not only pertain to the benefits system, but also the financial component of the recovery operation. Multiple participants expressed frustration at being addressed through financial settlement. For example:

*Money comes and goes… But I will never get back time.*

[P3]

*It just feels like you get 10.000 euros and you have to be happy with it. That’s all that you’re getting and shut up now.*

[P4]

These quotes capture the distrust participants still feel toward the government. Although the government has acknowledged its failure and financial compensation was awarded, it does not seem to help affected youth to come to terms with the benefits scandal. The emphasis on time, which was linked to a missed childhood by multiple participants, shows that the scandal’s harm was existential, not only economic.

Similarly, another participant emphasized that the recovery operation should focus on real help rather than symbolic gestures:

*Just look at what we missed out on and try to correct that by really improving our future.*

[P3]

Several participants also experienced the help offered by municipal services as not concrete enough:

*Nothing comes out. If you ever reach the point that you want help, [the municipal support organization says] ‘yes, but that we cannot do for you’*.

[P7]

##### From Coping to Contribution

Despite hardship, many of the participants emphasized how they were focused on a better future; for themselves, as well as other affected young people. Several participants were pursuing higher education, entrepreneurship, or community leadership roles, which is reflected in the semantic network (“stage” [internship] and “lopen” [to follow]; “diploma” [degree] and “halen” [to obtain]; and “studeren” [to study] and “recht” [law]). Remarkably, 3 of the 10 interviewees work for the municipality and/or tax services, while remaining distrustful toward these institutions. For example, one participant that worked as a legal worker for a governmental organization said:

*As an individual I feel a strong drive to help others. But at the same time I accept that I am just a pawn in a large organization.*

[P5]

This illustrates a nuanced position: critical of systems, yet willing to work within them to improve outcomes for others. This participant linked his drive for stability and achievement directly to the precariousness of his youth:

*The last few years were about survival. Creating stability, a roof above my head.*

[P5]

Several participants noted how they have received support from individuals or groups in their social networks. For example, participant 3 described how their religious community was the only one to believe and support him. It is for that reason that some of those participants now try to help others. Moreover, most of them describe that coming to terms with the scandal was intertwined with helping others. Participant 10 explained that supporting peers gave meaning to their own hardship. Participant 2 described how the experiences shaped them into someone who now actively helps others in his work, framing this as a way to turn hardship into purpose. This pattern of affected youth working hard for a better future to escape their past is also reflected in the semantic network, with links between “verleden” (past) and “toekomst” (future), and “betekenen” (to mean), “zoeken” (to search), “oplossing” (solution). For some, helping others appeared to be a coping mechanism: by guiding others, they reclaimed agency over their own narrative:

*Our (peer) group is a safe space. So if you feel the need to let your story out during a meeting, then that is possible.*

[P4]

### ToC

#### Overview

Across themes, youth framed the scandal in everyday terms. The results suggest that shame and fear of being judged help explain why youth remain out of sight, while distrust helps explain why engaging directly with the municipality feels like too big a step. The interviews suggested not a lack of need, but a lack of readiness and trust.

This suggests that a direct call-to-action to municipal supports is a bad fit. A staged pathway seemed more appropriate: first a private, noninstitutional entry point such as peer contact, followed by optional referral to municipal support. Youth who had largely come to terms with the scandal, and who expressed a desire to help others, could function as credible messengers for those who were still avoiding contact or felt stuck.

#### Segmentation

The interviews showed that some participants were oriented toward the future and already contributing to peers, while others described still being overwhelmed by the past or unable to imagine entering any municipal support process. These differences appeared central to how youth positioned themselves toward municipal support.

At the same time, we did not have quantitative data on the size of these subgroups. The municipality also did not have a complete overview of affected youth entitled to support, which was precisely why outreach through this project was deemed necessary. To structure these differences in readiness, we used Rogers’ Diffusion of Innovations theory [32] as a mental model. Our reasoning was that engaging with municipal support, or even acknowledging the need for support, can be seen as a step that not all youth are equally ready to take. The model offered a way to think about relative positioning without assuming exact numbers. As such, we distinguished 5 groups (Figure 3):

1. Affected youth unknown to the municipality.
2. Youth that are not ready to come to terms with the benefits scandal (Late majority and laggards).
3. Youth that are ready, but do not know how to come to terms with the benefits scandal (Early majority).
4. Youth that are ready to come to terms with the benefits scandal (Early adopters).
5. Youth that have come to terms with the benefits scandal and want to help others. (Innovators).

**Figure 3.**
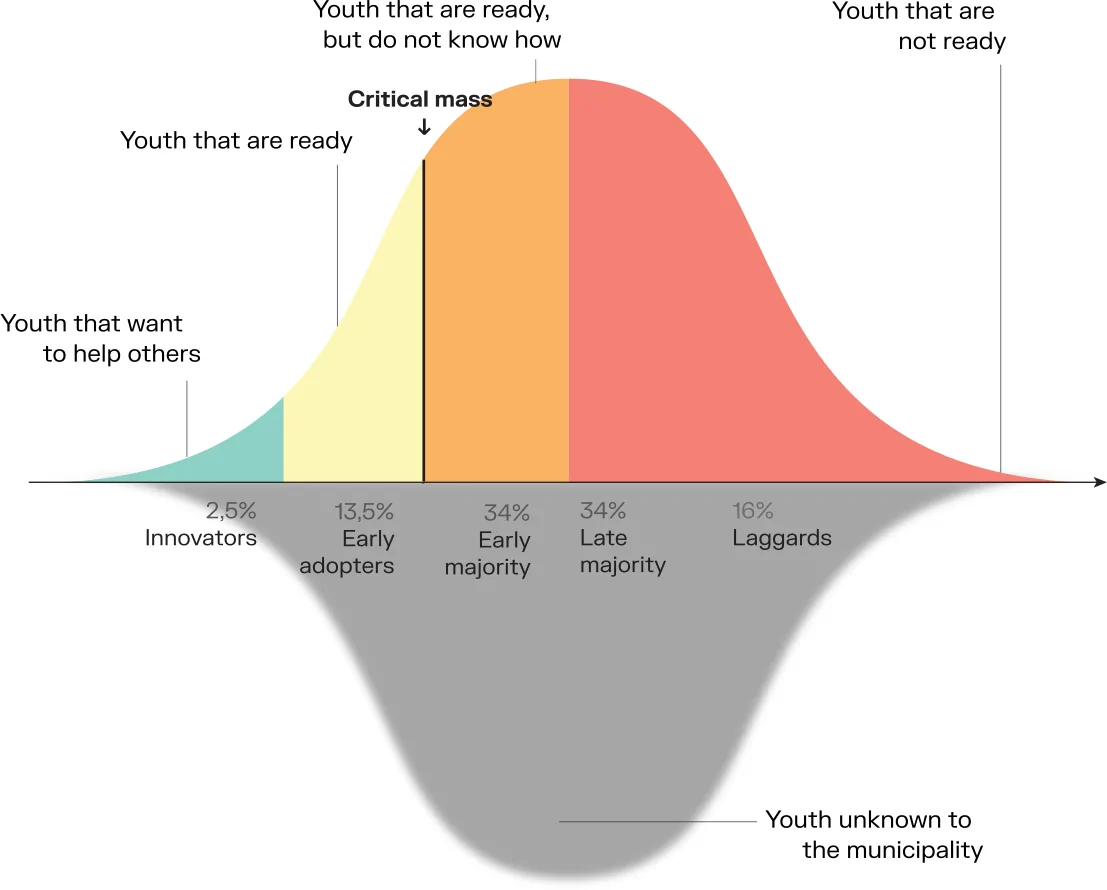
Readiness-based segmentation. Adapted from Rogers [32].

The intention was not to assign individuals rigidly to categories, but to design messages that could move youth one step forward. Those not ready can be approached with recognition and peer-based inspiration and entry points. Youth who were ready can be activated more directly. Youth who had processed their experiences and wanted to support others can be positioned as examples. Furthermore, the interviews suggested that once a sufficient number of such youth gathered in a shared network, their experience and energy could sustain the process. In that sense, the idea of a “critical mass” was not theoretical only, but grounded in participants who already described helping peers or creating safe spaces.

#### Means and Messages

Building on this segmentation, we developed a ToC that connected specific messages and intervention components to each stage of readiness (Figure 4).

1. The first step was Reach. The assumption was that entertainment media, in this case a rap track and music video, could organically reach affected youth, including those unknown to the municipality.
2. The second step was Acknowledge. The track and video explicitly recognized what had happened and articulated experiences in everyday language. The aim was that youth would feel heard and represented, thereby lowering the threshold to engage further.
3. The third step was Inspire. Through the narrative of the creator and the youth involved, as well as follow-up social media content and media appearances, the project sought to provide social proof that life after the scandal is possible.
4. The fourth step was Activate and Connect*.* Youth could explore options to address debts, missed education, or other consequences, and connect digitally with peers linked to the Flow Your Mind network (eg, via WhatsApp groups). This offered a low-threshold way to build trust before attending physical meetings.
5. Once connected to the Flow Your Mind network, peers could Help one another and, where appropriate, refer each other to municipal services through an individual track.
6. As youth regained stability, they could be Empowered to support others, for example by managing groups or acting as peer coaches.

Several assumptions underpinned this logic. First, that the chosen format and creator would be intrinsically appealing to affected youth and capable of organic reach. Second, that youth would indeed recognize themselves in the content and feel acknowledged. Third, that exposure to peers who had moved forward would have an encouraging effect. Before further scaling the online engagement and Flow Your Mind phases, these assumptions were tested during a pilot event.

**Figure 4.**
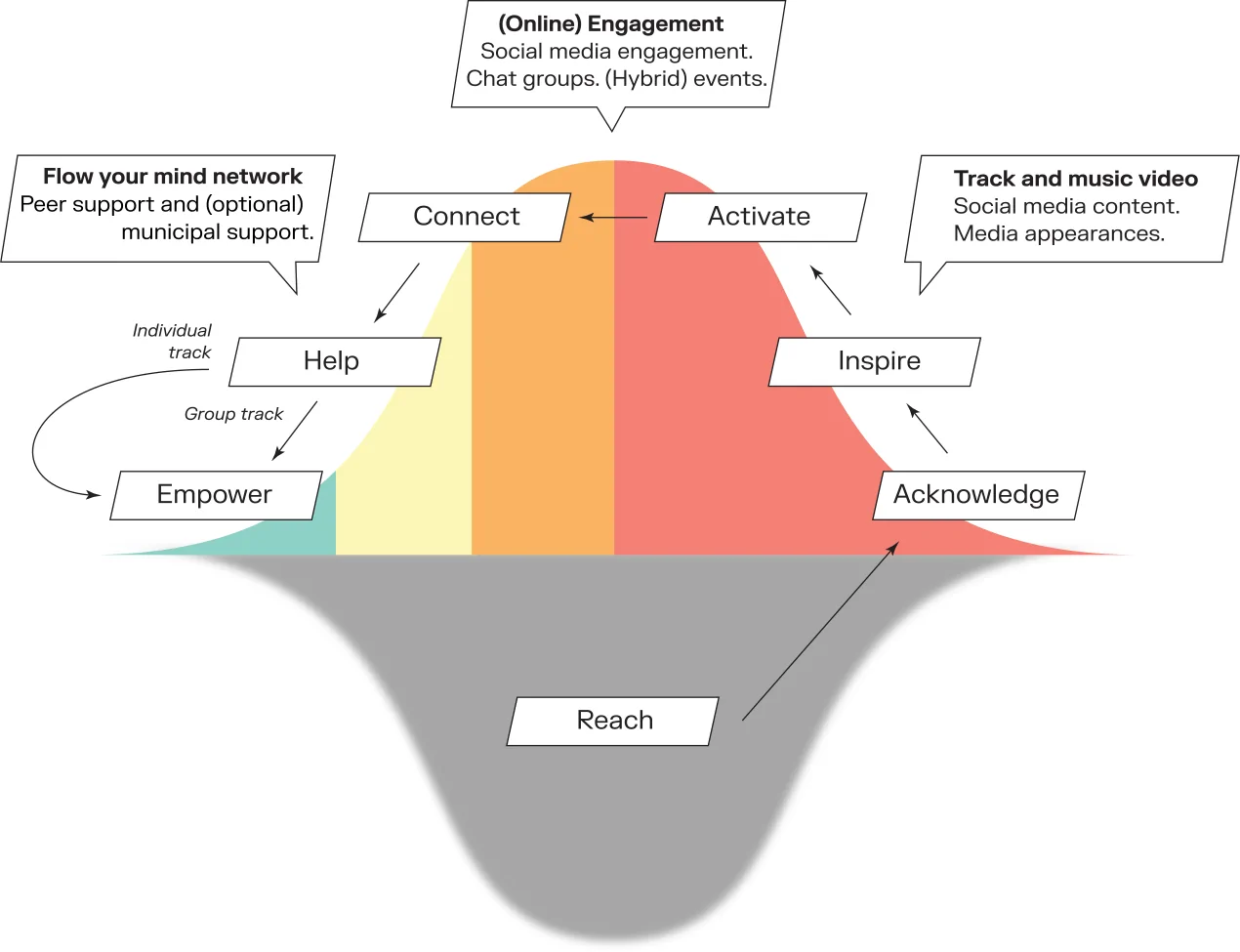
The theory of change, showing the core objective for each audience segment and the corresponding program components.

### Phase 2: Pilot Implementation

#### Cocreation

In line with the strategy, the track and video were meant to capture both (1) what young people went through during the benefits scandal, and (2) how they found ways to move forward. The structure of the lyrics reflects the intended balance between acknowledgment and forward movement (Multimedia Appendix 2). The first part focuses on acknowledging the past and present; the second part shifts towards strength, togetherness, and a brighter future.

#### Pretest

##### Overview

The 3 aforementioned assumptions about what the rap track and video were supposed to achieve were tested during a Flow Your Mind event in November 2023:

1. Whether the format and creator would feel intrinsically appealing and shareable (Reach*),*
2. Whether affected youth would recognize themselves and feel acknowledged (Acknowledge*),*
3. Whether exposure to the stories of peers who had moved forward would feel encouraging rather than distancing (Inspire*).*

Furthermore, we wanted to use the pilot event to explore whether social media engagement and (hybrid) events would be a low-threshold and appropriate way to connect youth to the Flow Your Mind network, and if so, what this should look like exactly in practice.

The first responses after showing the video were strong and emotional. During the Q&A, one of the affected young people that collaborated on the rap track and video said:

*I am glad that I worked on this. For others, because they may recognize themselves in my story. And for myself, because by talking about this I work on coming to terms with this whole situation.*

Youth in the audience spoke about being emotionally hit, about recognition, and about what the video could do if it reaches peers who are still out of sight. One participant captured it in simple terms:

*I think it takes away a piece of shame…precisely because it gets shared.*

Others immediately linked the video to connection (“not being alone”) and to practical next steps: using the video as a reason to talk to peers, ask questions, and exchange concrete tips. Against that background, we then looked more specifically at how the three assumptions held up.

##### Reach

Youth did not question whether the format fits their preferences; instead they talked about how it should spread and what needs to be in place when someone encounters it. This came back in the Q&A itself, where one of the youth involved in the production explicitly connected sharing to reaching others:

*I hope the video gets shared, and that people know: I’m not alone, and we do this together.*

During the group discussion, the dominant preference was that sharing should be paired with a low-threshold way to respond. Youth emphasized that affected peers should be able to reach out via DMs and then move into WhatsApp or Messenger groups, rather than being pushed straight to Flow Your Mind events or formal support.

##### Acknowledge

Reactions around acknowledgment were strong and concrete. Youth described the first part of the track as “confronting,” precisely because it mirrors “how it was at home,” and what that brought with it. At the same time, recognition was not framed as “staying in the past,” but as a necessary step to reduce isolation and taboo: “I’m not alone.”

In other words, youth did not respond to the video as an institutional message; they responded to it as something that speaks “normal language” about what happened, and makes it easier to talk. This matched what we were aiming for in the “acknowledge” step: not an institutional story, but everyday language that signals: ”this happened, and you’re not crazy for feeling the impact.*”*

##### Inspire

Youth responses suggested that inspiration did not come from “positivity,” but from seeing strength and options without denying what happened. In the pilot material, youth explicitly described recognizing themselves in the second part of the track, especially in the sense of pulling strength from each other and “getting through it together.”

This also surfaced when youth were asked what the video could trigger in others. One participant framed it as a prompt to think and act:

*The track makes you think again — what I can really do for myself now?*

Others connected inspiration directly to helping peers by exchanging practical knowledge (“together you know more”), and described the clip as a concrete motive to start those conversations.

Taken together, the pilot supported the staged logic from the ToC: the video can work as a credible entry point, but youth repeatedly stressed that the next step should stay low-risk and youth-controlled (eg, direct messaging or group chat first), with formal support as an option. These pilot findings informed the subsequent design of online engagement and follow-up activities.

### Evaluation

The pilot itself was evaluated positively by the municipality, and there was initial support to continue (Project Manager, Municipality of Rotterdam; email, January 30, 2024). However, continuation required institutional conditions that were not in place: internally, municipal capacity to provide follow-up support was limited, and externally, no formalized agreements had been secured with partner organizations. As a result, the municipality did not proceed with the proposed strategy to reach affected youth that they had not yet managed to reach, but chose to concentrate on strengthening peer-to-peer and municipal support for youth already connected to the Flow Your Mind network, while building an alliance of partner organizations.

## Discussion

### Principal Findings

This case report demonstrates how formative research and EE principles were used to develop and pretest a ToC-driven intervention in Rotterdam: a cocreated rap track and music video with affected youth. The goal was to explore whether narrative media rooted in youth language and peer dynamics could serve as a first step toward municipal support for young people impacted by the childcare benefits scandal.

Across the formative interviews, youth described the scandal as a family trauma, marked by chronic stress, financial hardship, disrupted education, and a sense of stagnation. Years of hardship fostered self-reliance and institutional distrust, suggesting that outreach that starts with policy language or bureaucratic procedures would likely fail. Instead, effective outreach should prioritize recognition of lived experiences, inspiration through peer narratives, and gradual, peer-led support. The pilot supported this: youth saw their stories reflected in the rap track and music video and were inspired by its portrayal of resilience, without denying the struggles.

### Responsibility and Repair

#### Overview

Throughout this case, we were repeatedly confronted with an ethical tension inherent to the municipal role in the recovery program: the benefits scandal was not something youth chose, caused, or could reasonably be expected to come to terms with. The responsibility for the consequences of the scandal lies with the institutions that created the harm. At the same time, the affected young people are the ones who still have to live their lives.

Therefore, the ToC used here should not be understood as “motivating youth to fix themselves,” but as lowering barriers that institutions have helped create: shame, distrust, and the sense that formal processes will not lead to concrete help. The goal of the strategy was not to shift responsibility onto youth, but to make it easier for youth to approach support on their own terms.

#### After the Pilot

By early 2024, the municipality of Rotterdam decided not to proceed with the proposed implementation strategy (Project Manager, Municipality of Rotterdam; email, January 30, 2024). This decision was not driven by a negative evaluation of the pilot: rather, continuation required a lack of municipal capacity to actually help the youth that could potentially be reached and activated through the intervention. This is important because it affects how the case should be interpreted: the pilot demonstrated the fit and promise of our approach but did not progress into implementation. In other words, this case provides evidence about the early steps (Reach, Acknowledge, Inspire, and a first low-threshold activation), but not about later steps that require organizational follow-through (connect, help, and empower).

Notably, the project did not end in silence: Perron14 initiated a public release event because the youth involved strongly felt the track needed to be released. The track can now be found online [34,36], but the release context was different from the intended strategy.

### Comparison With Prior Work

The use of a ToC in this case aligns with its role in IM as a tool to systematically plan and evaluate complex social interventions [30,31]. The ToC provided a structured way to articulate the desired outcomes, the steps needed to achieve them, and the assumptions underlying these steps, making it possible to test and refine the intervention [29,33].

Our case aligns with scholarly work positioning EE as a “site of discourse” [39] which has become particularly relevant in the social media era [40], where audiences increasingly create and shape streams of media content themselves. Here, the value of EE lies not only in the narrative itself but in the conversations and actions it catalyzes, whether in everyday conversations, peer group chats, or informal support networks. In our pilot, youth described the video as something that normalized discussing the scandal, empowering them to find peers and explore next steps on their own terms.

A second EE-relevant observation concerns the role of the producer-artist, as he did more than just “create content.” He functioned as a credibility anchor and facilitator: someone who could speak the language of the audience, translate interview material into lyrics that felt natural, and host the Q&A in a way that kept it grounded and emotionally safe. The pilot suggests that an established creator can lower thresholds in 3 linked ways: by reaching and attracting attention, by striking the right chord in tone and framing, and by starting online conversations. This aligns with the role that (online) creatives can play according to work about modern EE [19,20].

### Limitations

This case has clear limitations. Both formative phases were highly qualitative in nature, and the project never reached the phase where we could quantify reach or engagement, nor track longer-term outcomes such as sustained peer participation, help-seeking behavior, or successful referral to municipal services. However, 3 practical lessons stand out from this case.

First, because the producer-artist had a record deal with a major label, the track could not be released under his artist profile. This has a probable negative effect on reach and the ability to create resonance in the mainstream media (eg, radio, television, interviews, and social media content) that would otherwise have been an integral part of the strategy to reach youth. Future work that seeks to leverage the visibility of established artists or influencers should settle distribution rights and label constraints by approaching it as an early design issue.

Second, we learned that it is important to not make cocreation structurally dependent on vulnerable youth. Early in the project, we noticed several no-shows in the interviews and, later, during the cocreation trajectory. We solved this by making youth involvement meaningful, while not structurally dependent on it. Participation remained optional: the producer-artist could move forward with the insights from the interviews while keeping the door open for youth to rejoin later.

Last, we learned that institutional readiness matters as much as participant readiness. The municipality’s decision to stop after the pilot shows that you can design a credible entry point, but if the receiving system cannot absorb new demand, scaling becomes risky in practical and ethical ways. Although we remain confident in the stepwise formative research approach, we recommend future researchers to be aware of support, capacity, and budget across the entire pathway. A staged strategy only works if each step, including follow-up, is realistically resourced. The IM approach can actually help in laying bare such caveats.

### Conclusions

This case provides a detailed example of how formative research and EE principles can be used to develop and pretest a ToC-driven intervention that aims to engage hard-to-reach youth with institutional support services around a sensitive topic. The interviews and pretest suggest that outreach that starts in everyday language, captures youth’s lived experiences, and offers low-risk peer connection as a first step can be effective. The iterative, stepwise approach of IM and ToC allowed for continuous refinement, ensuring the intervention remained responsive to the youth voices while testing assumptions at each stage. Cocreated entertainment media can serve as a credible entry point to reduce shame, open conversations, and connect youth to peer networks and formal support.

## Supplementary material

10.2196/94223 Multimedia Appendix 1 Music Video “Excuses/Tegenwind” [Apologies/Headwind].

10.2196/94223 Multimedia Appendix 2 Rap track lyrics “Excuses/Tegenwind” [Apologies/Headwind].

## Abbreviations

DPIA: Data Privacy Impact Assessment
EE: entertainment-education
GDPR: General Data Protection Regulation
IM: intervention mapping
Q&A: question and answer
ToC: Theory of Change
WMO: Wet medisch-wetenschappelijk onderzoek met mensen
