# Supplementary material for: Youth-Centered Intervention to Lower the Threshold to Municipal Support and Help Youth Come to Terms with the Dutch Childcare Benefits Scandal: Case Report
Source: JMIR Form Res. 2026 Sep 30;10:e94223. doi: 10.2196/94223 (PMC13626553; doi:10.2196/94223)
Supplement: Supplementary file 1 [file formative-v10-e94223-s002.pdf]

Appendix A: Lyrics

“Excuses/Tegenwind” [Apologies/Headwind]

Dutch	English Translation
**Deel 1 (Erkennen)**	**Part 1 (Ackownledge)**
Nieuwsfragment:<br>“‘Jij bent een oplichter!’ Dat is het stempel dat de belastingdienst duizenden ouders gaf. Volledig onterecht. Ze moesten duizenden, tienduizenden en soms honderdduizenden terugbetalen en niemand hogerop geloofde in hun onschuld.”	*News Clip:*<br>“‘You are a fraudster!’ That’s the label the tax authority gave to thousands of parents. Completely unjustified. They had to repay thousands, tens of thousands, and sometimes hundreds of thousands, and no one in power believed in their innocence.”
Rapper:<br>Een affaire is wederzijds, maar zo ging het niet.<br>Gezinnen aan de grond na een blauwe brief.<br>De staat die is schuldig van schandaal,<br>burgers plukken ze kaal,<br>maar de daders blijven anoniem.	*Rapper:*<br>A affair[1] is mutual, but that’s not how it went.<br>Families brought to their knees by a blue letter.[2]<br>The state is guilty of scandal,<br>stripping citizens bare,<br>while the perpetrators remain anonymous.
Dit is niet meer terug te draaien als mijn kindertijd.<br>Gezin die ging kapot ik kon geen kind meer zijn.<br>Nog een rilling op mijn rug als de deurbel gaat,<br>en de trauma’s van mijn ma als ze de bus moet legen.	This can’t be undone, my childhood is gone.<br>Family fell apart, I couldn’t be a child anymore.<br>Still a shiver down my spine when the doorbell rings,<br>and the trauma of my mom when she had to empty the mail box.
News Clip:<br>Dit heeft mijn leven verwoest.	*News Clip:*<br>This has destroyed my life.
Rapper:<br>Er is geen geld die mij dat terug kan geven.<br>Dat ik van geluk kan spreken,<br>dat ik sta op beide benen,	*Rapper:*<br>No amount of money can give that back to me.<br>That I can speak of happiness,<br>that I stand on my own two feet,

[1] The word affair refers to the government coining the scandal as the *toeslagenaffaire* (benefits affair).
[2] Letters from the tax authority come in blue envelopes.

**Dutch**	**English Translation**
en het rust zou geven.	and that it would bring peace.
Als jullie zouden erkennen waar het mis is gegaan.	If only you would acknowledge where it went wrong.
Voor jullie lijk ik niks dan een naam.	To you, I'm nothing but a name[3].
Heb gestaan in het donker en het licht kon niet aan.	I stood in the dark and couldn't turn on the light.
Als je wat te zeggen hebt kijk me dan aan,	If you have something to say, look me in the eye,
kijk me dan aan!	look me in the eye!
Het zijn meer dan adressen die je pijn hebt gedaan.	It's more than addresses that caused you pain.
Wat hebben wij je misdaan?	What did we ever do to you?
Ik heb niets in het leven gekregen behalve mijn naam.	I've been given nothing in life but my name.
En precies dat gebruik jij tegen mij,	And that's exactly what you use against me,
de excuses zijn wederzijds.	the apologies are mutual.
Zangeres:	*Singer:*
Sorry dat jij dit land moet dragen,	Sorry that you have to carry this country,
zijn er nog vragen zijn er nog vragen?	are there any questions left? Are there any questions?
Kom hier en vraag ze.	Come here and ask them.
Maar of je antwoord krijgt? Dat weten zij…	But will you get an answer? They know…
Ik zei zijn er nog vragen?	I said, are there any questions?
Kom hier en vraag ze,	Come here and ask them,
maar of je antwoord krijgt?	but will you get an answer?
**Deel 2 (Inspireren)**	**Part 2 (Inspire)**
Rapper:	*Rapper:*
Er gebeurt een hoop.	A lot is happening.
Maar er is nog hoop.	But there is still hope.
En ik zat in nood,	And I was in need,
maar mij krijg je niet klein,	but you won't break me,
want we dromen groot.	because we dream big.
Zoek jij een vriend? Ik ben je lotgenoot.	Are you looking for a friend? I am your peer.
Samen varen op die boot.	Sailing together on that boat.
Tot er land in zicht is.	Until land is in sight.

[3]This refers to most of the victims having a non-Dutch last name

Dutch	English Translation
Dit gevoel ik mis dit.	I miss this feeling.
Rapper en zangeres:<br>Zolang ik weet waar ik naartoe ga, hoef je mij niet te vertellen wat de reden is.<br>En dingen blijven gaan zoals ze gaan, maar ik blijf rennen naar de zon terwijl het regen is.	*Rapper and Singer:*<br>As long as I know where I'm going, you don't need to tell me the reason.<br>And things keep going as they do,<br>but I keep running towards the sun while it's raining.
Zolang je maar gelooft in mij!<br>Alles valt op z'n plek,<br>ze namen meer dan dat ik heb.<br>Maar dit wordt ooit verleden tijd!<br>Alle vragen die ik heb,<br>en wat krom is trek ik recht.	As long as you believe in me!<br>Everything will fall into place,<br>they took more than I have.<br>But this will someday be in the past!<br>All the questions I have,<br>and what is crooked, I will straighten.
Want jij gelooft in mij!<br>Ook als het tegenzit.<br>Samen gaan we hier doorheen,<br>blijven wij hier staan,<br>ook met tegenwind!	Because you believe in me!<br>Even when things go wrong.<br>Together we will get through this,<br>we will stay standing here,<br>even against the wind!
Rapper:<br>Ook met tegenwind,<br>blijf ik enigszins hier in evenwicht, yeah!<br>Niet uit het veld te slaan, nog steeds. . .<br>hobbels op de weg,<br>ik blijf op dreef,	*Rapper:*<br>Even against the wind,<br>I remain somewhat balanced here, yeah!<br>Can't be knocked down, still. . .<br>bumps on the road,<br>I keep going,
Ik leef!	I live!
Voor mezelf en niet voor een ander.<br>Geen dader maar slachtoffer, dit heeft mij veranderd.<br>Ondanks dat ligt mijn lot in mijn eigen handen.<br>Als je hebt geloofd in mij,<br>bij deze: ik dank je!	For myself and not for someone else.<br>Not a perpetrator but a victim, this has changed me.<br>Despite that, my fate is in my own hands.<br><br>If you have believed in me,<br>consider this my thanks!
Rapper en zangeres:<br>Zolang ik weet waar ik naartoe ga, hoef je mij niet te vertellen wat de reden is.<br>En dingen blijven gaan zoals ze gaan, maar ik blijf rennen naar de zon terwijl het regen is.	*Rapper and Singer:*<br>As long as I know where I'm going, you don't need to tell me the reason.<br>And things keep going as they do,<br>but I keep running towards the sun while it's raining.

**Dutch**	**English Translation**
Zolang je maar gelooft in mij!<br>Alles valt op z'n plek,<br>ze namen meer dan dat ik heb.<br>Maar dit wordt ooit verleden tijd!<br>Alle vragen die ik heb,<br>en wat krom is trek ik recht.	As long as you believe in me!<br>Everything will fall into place,<br>they took more than I have.<br>But this will someday be in the past!<br>All the questions I have,<br>and what is crooked, I will straighten.
Want jij gelooft in mij!<br>Ook als het tegenzit.<br>Samen gaan we hier doorheen,<br>blijven wij hier staan,<br>ook met tegenwind!	Because you believe in me!<br>Even when things go wrong.<br>Together we will get through this,<br>we will stay standing here,<br>even against the wind!
